# An efficient framework for obtaining the initial cluster centers

**DOI:** 10.1038/s41598-023-48220-3

**Published:** 2023-11-27

**Authors:** B. K. Mishra, Sachi Nandan Mohanty, R. R. Baidyanath, Shahid Ali, D. Abduvalieva, Fuad A. Awwad, Emad A. A. Ismail, Manish Gupta

**Affiliations:** 1 Silicon Institute of Technology, Bhubaneswar, Odisha 751024 India; 2 School of Computer Science & Engineering (SCOPE), VIT-AP University, Vijayawada, Andhra Pradesh 522237 India; 3 School of Electronics Engineering, Peking University, Beijing, China; 4 Doctor of Philosophy in Pedagogical Sciences, Tashkent State Pedagogical University, Bunyodkor Avenue, 27, 100070 Tashkent, Uzbekistan; 5 Department of Quantitative Analysis, College of Business Administration, King Saud University, P.O. Box 71115, 11587 Riyadh, Saudi Arabia; 6 Division of Research and Technology, Lovely Professional University, Phagwara, India

**Keywords:** Computational biology and bioinformatics, Health care, Engineering, Mathematics and computing

## Abstract

Clustering is an important tool for data mining since it can determine key patterns without any prior supervisory information. The initial selection of cluster centers plays a key role in the ultimate effect of clustering. More often researchers adopt the random approach for this purpose in an urge to get the centers in no time for speeding up their model. However, by doing this they sacrifice the true essence of subgroup formation and in numerous occasions ends up in achieving malicious clustering. Due to this reason we were inclined towards suggesting a qualitative approach for obtaining the initial cluster centers and also focused on attaining the well-separated clusters. Our initial contributions were an alteration to the classical K-Means algorithm in an attempt to obtain the near-optimal cluster centers. Few fresh approaches were earlier suggested by us namely, far efficient K-means (FEKM), modified center K-means (MCKM) and modified FEKM using Quickhull (MFQ) which resulted in producing the factual centers leading to excellent clusters formation. K-means, which randomly selects the centers, seem to meet its convergence slightly earlier than these methods, which is the latter’s only weakness. An incessant study was continued in this regard to minimize the computational efficiency of our methods and we came up with farthest leap center selection (FLCS). All these methods were thoroughly analyzed by considering the clustering effectiveness, correctness, homogeneity, completeness, complexity and their actual execution time of convergence. For this reason performance indices like Dunn’s Index, Davies–Bouldin’s Index, and silhouette coefficient were used, for correctness Rand measure was used, for homogeneity and completeness V-measure was used. Experimental results on versatile real world datasets, taken from UCI repository, suggested that both FEKM and FLCS obtain well-separated centers while the later converges earlier.

## Introduction

In every aspect of our day to day requirements it is often necessary to sensibly organize data into their relevant groups. This not only gives clarity about their whereabouts but also helps us to pick them from their respective assemblage much faster. So, the most important thing that needs to be considered is the correct grouping among them. Consequently, in order to expedite the retrieval of relevant information from a group or sub-group, numerous consistent practices have been developed, one of which is data clustering (Odell and Duran ^1^). The main goal is to create divisions for the whole data set into reasonably smaller homogenous subdivisions so that the objects present in a subgroup will be having similar characteristics with each other and reasonably differ from those present in other subgroups.

For any clustering technique to come to practice, the first and foremost step is the selection of initial cluster centers which defines the number of clusters to be created. There are various ways in which the initial cluster centers are initialized. This initialization plays a crucial role for the end result of clustering. Random selections of the initial centroids are preferred by many clustering algorithms. This adds to the simplicity of the approach and also to the computation time of the algorithm. When the initial centroids are chosen randomly, roughly no time is spent on their selection step, which lessens the overall execution time as well as the time complexity of the algorithm. However, with random initialization of centroids, different runs will produce different clustering results. Sometimes, the result will show excellent subgroups formation while in most cases the resulting clusters are often poor. It is possible to obtain an optimal clustering when the two randomly selected initial cluster centers fall somewhere in a pair of clusters, because the cluster centers will reorganize among themselves, one to each cluster. Unfortunately, in some cases if the number of clusters is more, it is more and more likely that in any case one pair of clusters will have merely one initial cluster center. In this case, since the pairs of clusters are farther apart than clusters within a pair, the clustering algorithm will fail to reallocate the centroids among the pairs of clusters, and simply local minima will result. In other words, empty clusters may be achieved if no points are allocated to a cluster during the assignment phase of clustering. “Because of the problems with using randomly selected initial centroids, which even repeated runs may not overcome, there is an need to develop some ways for better initialization of initial center of clusters.”

In most cases it is found the clustering model designed for the purpose faces difficulties in identifying the “natural clusters”. These cases arise when the clusters have widely different shapes, sizes and compactness. For example, while considering a dataset to be grouped into three clusters, if one of the clusters formed is relatively bigger than the other two, the bigger cluster is broken down and one of the smaller clusters is combined with a section of the bigger one. In another instance, a clustering model may fail to create well separated subgroups when the two smaller clusters are much compact than the bigger one and obviously lead to inaccurate conclusions about the structure in the data. “There is a need to design a clustering model which will be efficient enough to perform the required formation of subgroups with improved reliability and accuracy and more importantly achieving the result much faster with varying datasets”.

These are the two motivational factors which gave us a direction to continue our work in this aspect.

Although, we as individuals are exceptional cluster seekers, but there is a necessity of excellent clustering algorithms which can operate on versatile data sets and provide us with effective cluster formation. A bunch of clustering ensemble methods proposed by eminent researchers has been projected over the last few years to present a solution for selection of initial cluster centers as well as obtaining good cluster formation. A few of those are discussed here.

Na et al.^2^ conducted an examination of the constraining facets of the K-Means algorithm, proposing an alternative approach for assigning data points to distinct clusters. Their method mitigates the computational time required for K-Means. In a comprehensive survey, Xu et al.^3^ scrutinized a diverse array of clustering methodologies along with their practical applications. Additionally, they deliberated on various proximity criteria and validity metrics that influence the resultant cluster configuration. Cheung^4^ introduced a tailored K-Means variant capable of achieving precise clustering without the need for initial cluster assignments. This technique demonstrates notable efficacy in clustering elliptically-shaped data, a pivotal aspect of the research. In an innovative endeavor, Li^5^ advocated for the adoption of the nearest neighbor pair concept in determining the initial centroids for the K-Means algorithm. This method identifies two closely neighboring pairs that exhibit significant dissimilarity and reside in separate clusters. This represents one among several approaches aimed at advancing the determination of initial cluster centroids. Nazeer et al. ^6^ proposed a further progression towards ascertaining nearly accurate initial centroids and subsequently assigning objects to clusters, albeit with the stipulation that the initial number of clusters must be specified as input. In order to mitigate the stochastic selection of initial cluster centers in the K-Means algorithm, Cao et al.^7^ introduced a model wherein the cohesion degree within a data point's neighborhood and the coupling degree among neighborhoods are defined. This model is complemented by a novel initialization technique for center selection.

Kumar et al.^8^ suggested a kernel density-based technique to determine the initial centers for K-Means. The plan is to pick an initial data from the denser part of the data set since it actually reflects the characteristics of the data set. By doing this the presence of outliers are avoided. The performance of their method was tested on different data sets using various validity indices. The result showed that the given method has superior clustering performance over the traditional K-Means and K-Means++ algorithm. Kushwaha et al.^9^ proposed a clustering technique based on magnetic strength with an objective to locate the best location of centroids in their respective clusters. Data generate force directly to magnetic force and the best possible position for centroids is when the force by all data draws nearer to zero. Results from experiments imply that the suggested method getaway from local optima. However, it’s only limitation is, it needs to have prior information of the number of clusters to be created. Mohammed et al.^10^ introduced WFA selection, a modified weight-based firefly selection algorithm designed to attain optimal clusters. This algorithm amalgamates a selection of clusters to generate clusters of superior quality. The results demonstrate that this algorithm yields newly condensed clusters when compared to a subset of alternative approaches.

In a recent study, Fahim^11^ conducted a comprehensive review of the classical Density-based spatial clustering of application with noise (DBSCAN) algorithm, scrutinizing its inherent limitations and proposing a method to mitigate them. The suggested technique identifies the maximum allowable density level within each cluster, enabling DBSCAN to evaluate clusters with varying densities. Comparative analysis confirmed the effectiveness of the proposed method in accurately determining the actual clusters. Fahim^12^ suggested a technique to discover an optimal value for ‘k’ and initial centers of K-means algorithm. A pre-processing step is used for this purpose before K-means is applied. Density-based method is used for this purpose as it does not need to initially mention the number of clusters and also it calculates the mean of data in each cluster. The suggested method also uses the DBSCAN algorithm as a pre-processing step. Experimental data suggested that, this method ultimately improves the final result of clustering and also reduces the number of iterations of K-means. Khandare et al.^13^ presented a modification to the K-Means and DBSCAN clustering algorithms. Their proposed approach enhances clustering quality and establishes well-organized clusters through the incorporation of spectral analysis and split-merge-refine methods. Notably, their algorithm addresses the minimization of empty cluster formation. Experimental assessments were conducted, taking into account parameters such as cluster indices, computation time, and accuracy, on datasets of diverse dimensions.

Yao et al.^14^ grouped the non-numeric attributes present in the dataset according to their properties and discovered the analogous similarity metrics in that order. They proposed a method for finding the initial centroids based on the dissimilarities and compactness of data. Once the centers were obtained, clustering was performed on modified inter-cluster entropy for miscellaneous data. Results concluded that since the initial centers determined were optimal so it resulted in good clustering accuracy rate (CAR). Ren et al.^15^ suggested a two-step structure for scalable clustering in which the initial step determines the frame structure of data and the final step does the actual clustering. Data objects are initially placed across a 2-D grid and are clustered using different algorithms, each giving a set of partial core points. These points correspond to the dense parts of data, which form centers for center-based, modes for density-based or means for probability-based types of clustering. This method can speed-up the computation and produces robust clustering. Results have shown the usefulness of the method.

Franti et al. ^16^ suggested a better initialization technique that improves the clustering efficiency of K-means. When there are overlapping clusters, using farthest point heuristic, malicious clusters may be reduced from 15 to 6% and when the method was repeated for 100 times, a further reduction to 1% was noted. However, they remarked that dataset with well separated clusters depends mostly on proper initialization of centers.

Mehta, et al.^17^ discussed and analyzed several proximity measures and suggested a way for choosing a proximity measure that can be used in hierarchical and partitioned clustering. They concluded that the average performance of clustering changes when diverse proximity measures were implemented. Mehta et al.^18^ further researched in document clustering in text mining and proposed a method by hybridizing the statistical and semantic features. The technique uses a fewer number of features but this hybridization improves the textual clustering and provides better precision within acceptable time limit.

Shuai^19^ proposed an improved feature selection and clustering framework. This model initially does the data processing and then uses the feature selection to obtain important features from the dataset. This was followed by hybridizing K-means and SOM neural network to perform the actual clustering. Finally, collaborative filtering was used to cluster datasets which constituted missing data to make sure that all samples can acquire results. Results obtained showed high accuracy in clustering and interpretability. Nie et al.^20^ proposed a clustering technique where there is no need to calculate the cluster centers in each iteration. In addition, the proposed technique provides an efficient iterative re-weighted approach to solve the optimization problem and shows a faster convergence rate.

Ikotun et al.^21^ broadly presented a summary and taxonomy of the widely used K-means clustering algorithm and its different variants created by various researchers. The record of K-means, recent developments, different issues and challenges, and suggested potential research viewpoint are discussed. They have found most of the research work has been carried out on solving the initialization issues of K-means however, very little focus has been given on addressing the problem of mixed data type. This survey will help practitioners to work on this aspect.

## Methods

One of the major drawbacks of traditional K-Means approach is the initial center selection which is done randomly. Random selection of centers may perhaps result in incorrect creation of clusters. Due to this issue, there are few suggestions mentioned in this work with an aim to minimise this limitation. In addition to this, there are suggestions for reducing the time complexity, actual computation time and the convergence criteria of the projected methods. Subsequently, all the methods are evaluated to verify how good each of them creates the subgroups. The methods for choosing the initial cluster centers are discussed below:

### Method-1: K-means

This is an unsupervised learning algorithm (Mac Queen^22^) to find K sub-groups from a given set of data, where the value of K is defined by the user. Initially, random K data points are selected as cluster centers. Each data point is assigned to its nearest cluster center to form disjoint sub-groups. Then the cluster centers are overwritten with the values obtained by taking mean of the data points present inside it. This process of reassigning data points to the nearest center and updating the cluster centers is repeated until there are no changes to the centers. The steps followed in K-means are:

- Take random K data points as initial centers
- Assign each data to nearest cluster center
- Update cluster centers by taking mean
- Repeat steps 2–3 until convergence

K-Means is the simplest way for getting different sub-groups of a given input. However, taking K random centers in step-1 makes it unpredictable. Every time this algorithm is executed it produces different results. So there is no precision in the clustering outcome. Even there is a possibility that at times it may result in creation of empty clusters.

It has an indefinite time complexity, since it is computationally a NP-hard problem. Step-2 of the procedure may repeat for an uncertain amount of time. However, if we constraint the clustering loop to a fixed value i, such that i < n, then the complexity of the algorithm will become O (i * K * n * d), where K and n are the number of clusters and data points respectively and d is the dimension of each data. Practically, the values of K and dare fixed and significantly smaller than n, hence the complexity is O (n).

### Method-2: far efficient K-means (FEKM)

**Figure 1:**
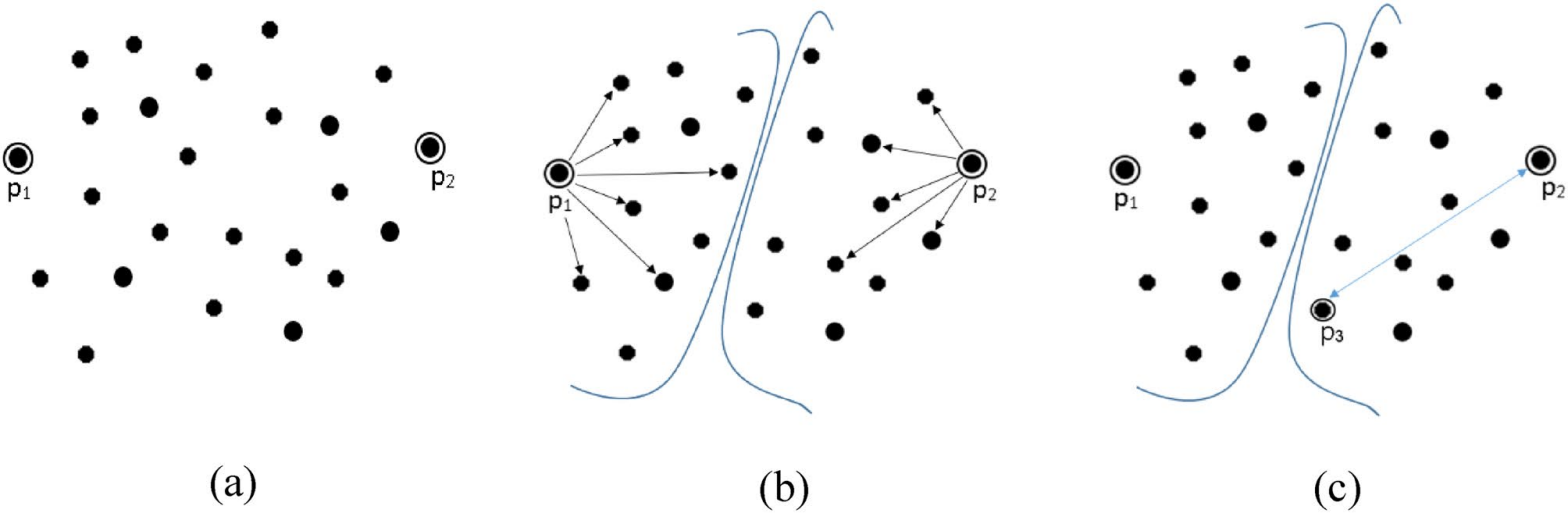
Computation of centers (**a**) farthest pair p_1_–p_2_ are two initial centers (**b**) distance of each point to its nearest center calculated (**c**) p_3_ is selected as third center since it is at maximum distance from its nearest center.

To overcome the limitations of selecting the initial cluster centers randomly, Mishra et al.^23^ suggested an innovative approach. The central idea of their approach is to obtain distant points as initial center which will result in disjoint, tight and precise cluster formation. The pseudo code representation of algorithm is as follows:
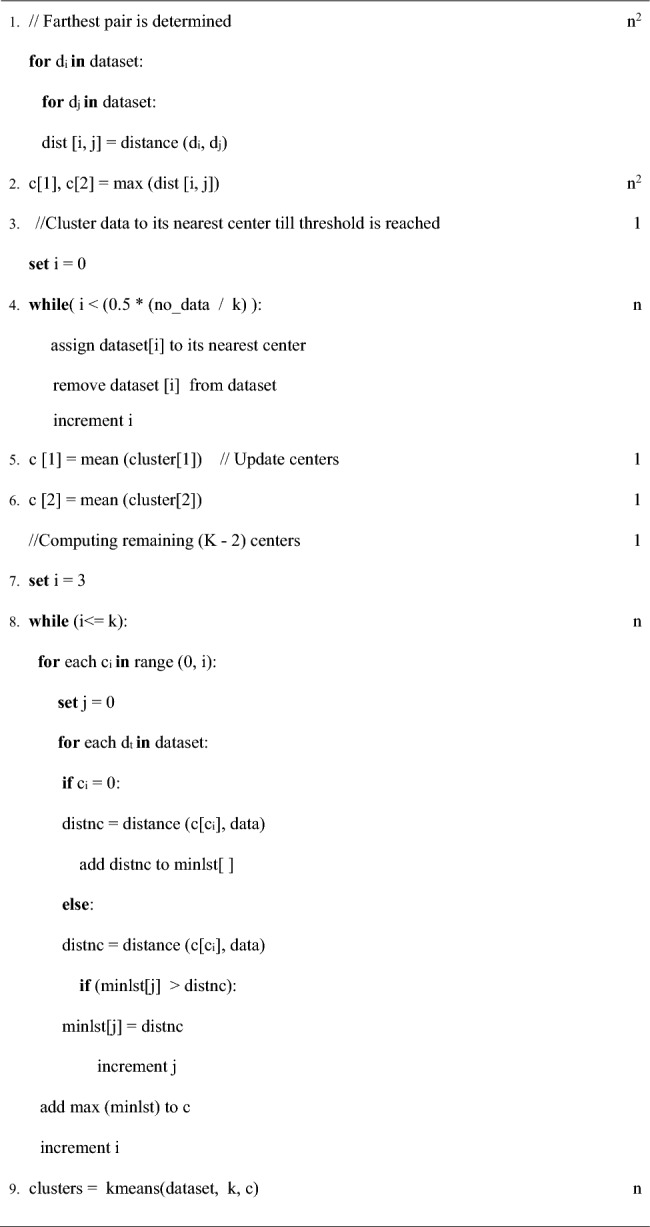


The algorithm works as follows. Distance of each data point to all other points present in the data set are computed and the data pair which lies farthest from each other are considered as first two initial centers in step 1 and step 2. Step 4 assigns data points to their nearest center until a pre-defined threshold value is reached. In step 5 and 6 centers are updated by taking mean of the partial clusters formed in previous step. The remaining K−2 centers are computed in step 8. The process of obtaining the centers is illustrated in Fig. 1.

Due to brute force comparisons between all the data points to find the farthest pair in Step 1, the loop has to run n^2^ times making its complexity Ɵ (n^2^). Hence, the overall complexity of FEKM is Ɵ (n^2^). The major drawback of FEKM is its worst case running time complexity which is Ɵ (n^2^). Methods like FEKM with quadratic time complexity are feasible for small data sets.

### Method-3: modified center K-means (MCKM)

Considering the quadratic time complexity of FEKM, (Mishra et al. IJISA^24^) suggested MCKM for selecting the initial cluster centers with complexity less than Ɵ (n^2^). The idea is to sort the data points with respect to a fixed point of reference, which is the last data as present in the data set matrix. Then divide the data set into K equal subgroups and compute the mean of each group to find initial centers. The procedure is presented as a pseudo code which is as follows:
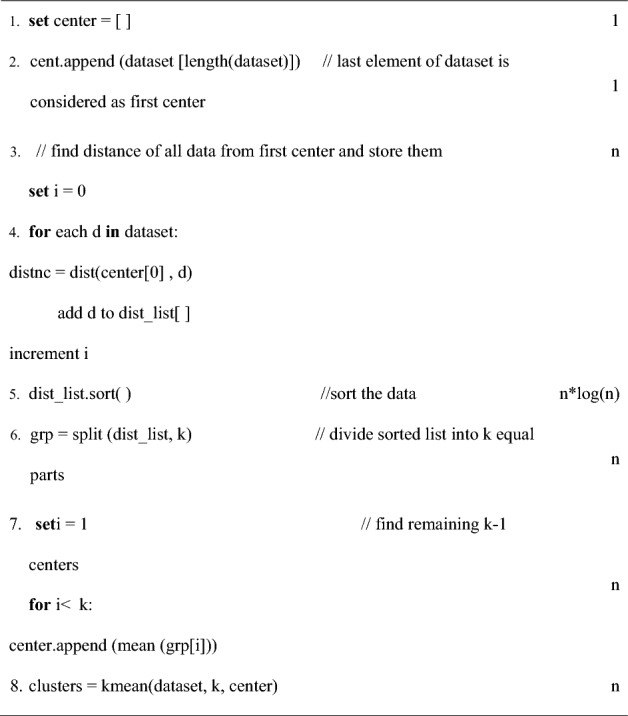


Step 2 sets the very last element present in the data set matrix as the first center which is the point of reference. In step-4, distance of each element is determined from the point of which makes its complexity O (n). Step 5 sorts all data using a sorting technique of complexity O (n* log (n)). In step 6, the sorted list is splitted into K equal subgroups which will cause the loop to run n times making its complexity as Ɵ (n). The centers are updated and mean of each group gives the rest k-1 center in step 7 whose complexity is Ɵ (n). Thus, overall complexity of MCKM is O (n* log (n)). This method is able to provide a systematic and efficient procedure to obtain initial centers. Unlike FEKM it has a better running time complexity. However, FEKM has an upper hand over MCKM in obtaining distant initial centers which results in better cluster formation.

### Hastening FEKM by constructing convex hull

FEKM uses brute force technique to find the farthest data pair and considers them as the first two initial centers. This results in quadratic time complexity of the algorithm. So, in order to reduce the complexity of FEKM, the concept of convex hull (Cormen^25^) is used. Convex hull is the smallest convex polygon that encloses all the data points of a given data set. These points are selected in such a way that, there exist no other points which remains outside the hull. By computing the convex hull, it is only required to compare the data points which form the vertices of the hull instead of considering every data points to obtain the farthest pair. There are two approaches considered in this research to achieve the farthest centers using convex hull. They are as follows:

### Farthest centers using graham scan (FCGS)

Graham scan approach (Graham^26^) maintains a stack which contains only the vertices of the hull. The procedure pushes all the data points into the stack and ultimately pops the point that is not a vertex. The distance between each vertex is determined to obtain the farthest pair to be considered as first two centers. Hence, it is not required to compare the distance between all other points. This reduces the complexity of the algorithm. The algorithm for FCGS is as follows:
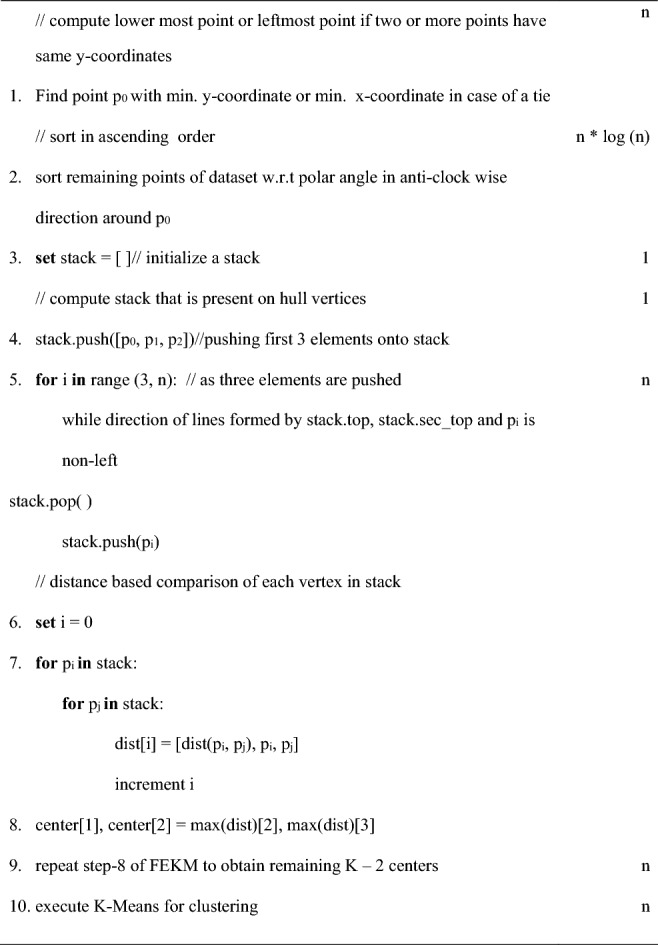


Step 1 of the algorithm chooses a point p_0_ with smallest y-coordinate value or the leftmost x-coordinate value if two or more points have equal y-coordinates. For this step 1 need to traverse to reach every data points which make its complexity Ɵ (n). In step 2 data points excluding p_0_ are sorted as per the polar angle around p_0_ in anti-clockwise order using a sorting algorithm of complexity O (n * log (n)). First three points of the sorted data set i.e. p_0_, p_1_, p_2_ are pushed into a stack in step 4. In each iteration of step 5, a data point is pushed into the stack and the orientation (Cormen^25^) formed by top three elements of the stack is checked. If the orientation is clock wise or non-left then pop( ) operation is performed. As it traverses n−3 points, step 5 has a complexity of O (n). After obtaining the stack of hull vertices, each vertex is distance-wise compared to find the farthest pair in step 7. Assuming number of elements in stack is m, the complexity of step 7 is Ɵ (m^2^). The remaining k−2 centers are computed in step 9 following FEKM. As m will be always less than n, overall complexity of FCGS is O (n * log(n)). Therefore, this method is able to obtain initial centers which will boost clustering performance with a reduced complexity of O (n * log(n)).

However, Graham scan is designed only for data sets with two attributes. If the dataset consists of more than two attributes then the algorithm fails because there are multiple values of polar angles. Dimensionality reduction is a way but may increase the running time of the algorithm drastically. Now, one of the solutions of FCGS is by using a method which can construct the convex hull in multiple dimensions. Modified FEKM using Quick hull is a solution to this aspect.

### Modified FEKM using Quickhull (MFQ)

**Figure 2:**
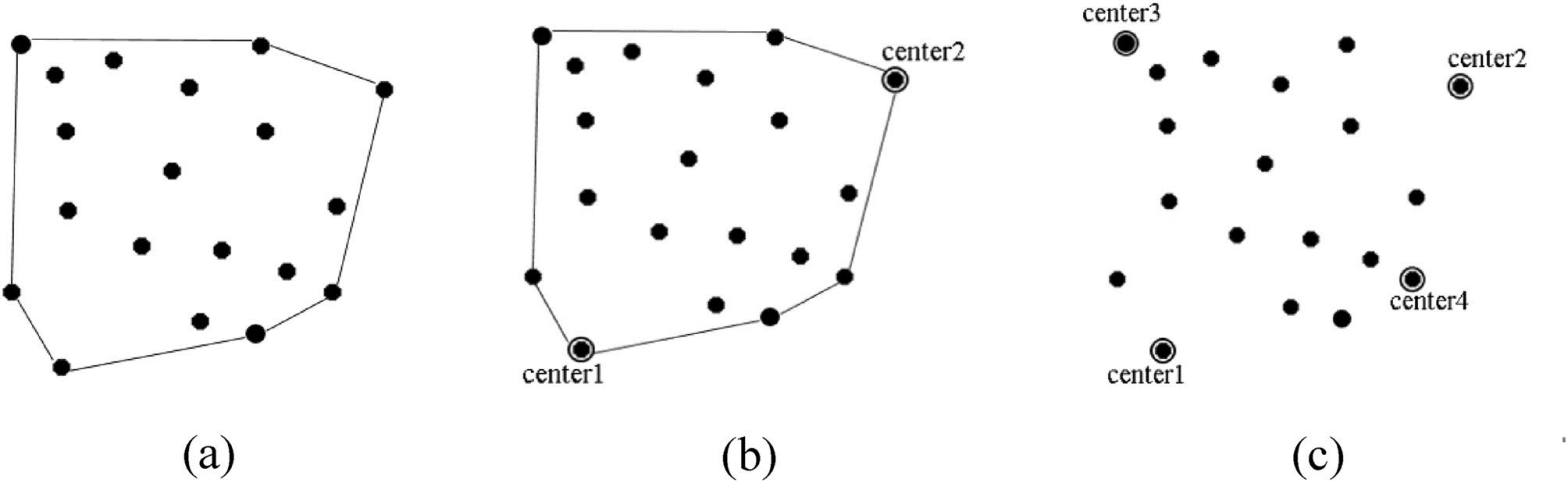
Illustration of MFQ (**a**) Step 1–7 of the algorithm is used to construct convex hull (**b**) Step 8 of algorithm compares all the vertices of the hull to find farthest pair as first two initial centers (**c**) Step 8 of FEKM is used to find remaining K−2 centers.

Quickhull proposed by (Bradford Barber^27^) may be used to construct convex hull for n-dimensional data. It computes the convex hull in a divide and conquer approach recursively. The pseudo code of algorithm is as follows:
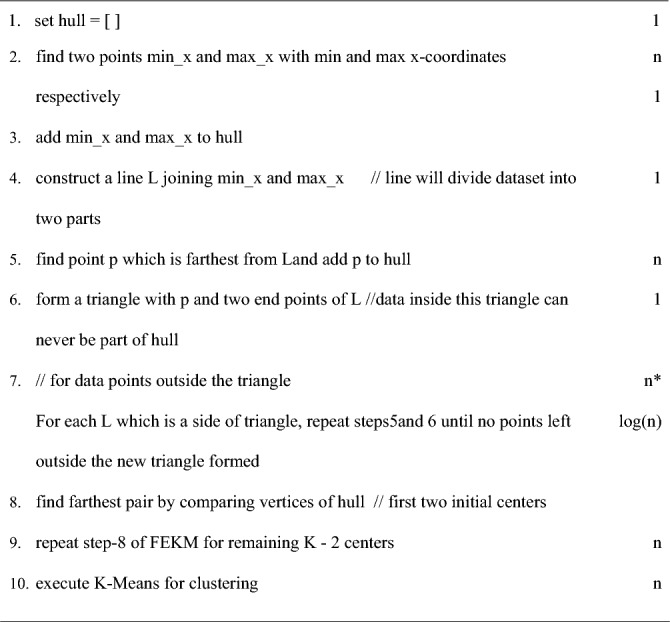


First seven steps of the algorithm are used to construct a convex hull. In this method, a list named hull is declared which store the vertices of the convex hull. The points with maximum and minimum x coordinate values are assigned to variables max_x and min_x respectively. A line L is constructed by joining min_x and max_x. This line will divide the data sets into two parts. A point p is found which is farthest from L and is added to hull. A triangle is formed by joining p and two end points of L. The points lying inside the triangle are not considered for further construction of the hull. For the remaining points present outside the triangle, each side of the triangle is assigned as L and steps5& 6of the algorithm are repeated recursively until there are no points left outside the last triangle formed. After obtaining the convex hull, the vertices lying on it are compared with each other to find the farthest pair, which is considered as first two initial cluster centers. Finally, step8 of FEKM is repeated to obtain K−2 remaining centers. The above procedure is illustrated in Fig. 2.

Step 2 of the method costs O (n) for traversing the data set to find minimum and maximum x-coordinate values. Step 5 computes a point farthest from line L making its complexity O (n). Step 5 and 6 is repeated recursively until no points are left outside the new triangle formed. The worst case complexity of Quickhull is O (n * log (n)) if dimension of the data is less than or equal to three. When the number of attributes is more than three then the complexity of this method increases. Next step8 of FEKM is called to determine the remaining K-2 initial centers which make its complexity O(n). So, unlike FCGS this method is able effectively operate on datasets with more number of attributes with a worst case of O (n * log (n)).However, the worst case complexity of this algorithm may exceed O (n^2^) when we have large number of attributes in data sets. In these cases, this method will prove to be inefficient than FEKM. For this reason, we suggest a method called Farthest Leap Center Selection (FLCS) to compute the farthest data pair in less than quadratic time complexity.

### Proposed method: farthest leap center selection (FLCS)

**Figure 3:**
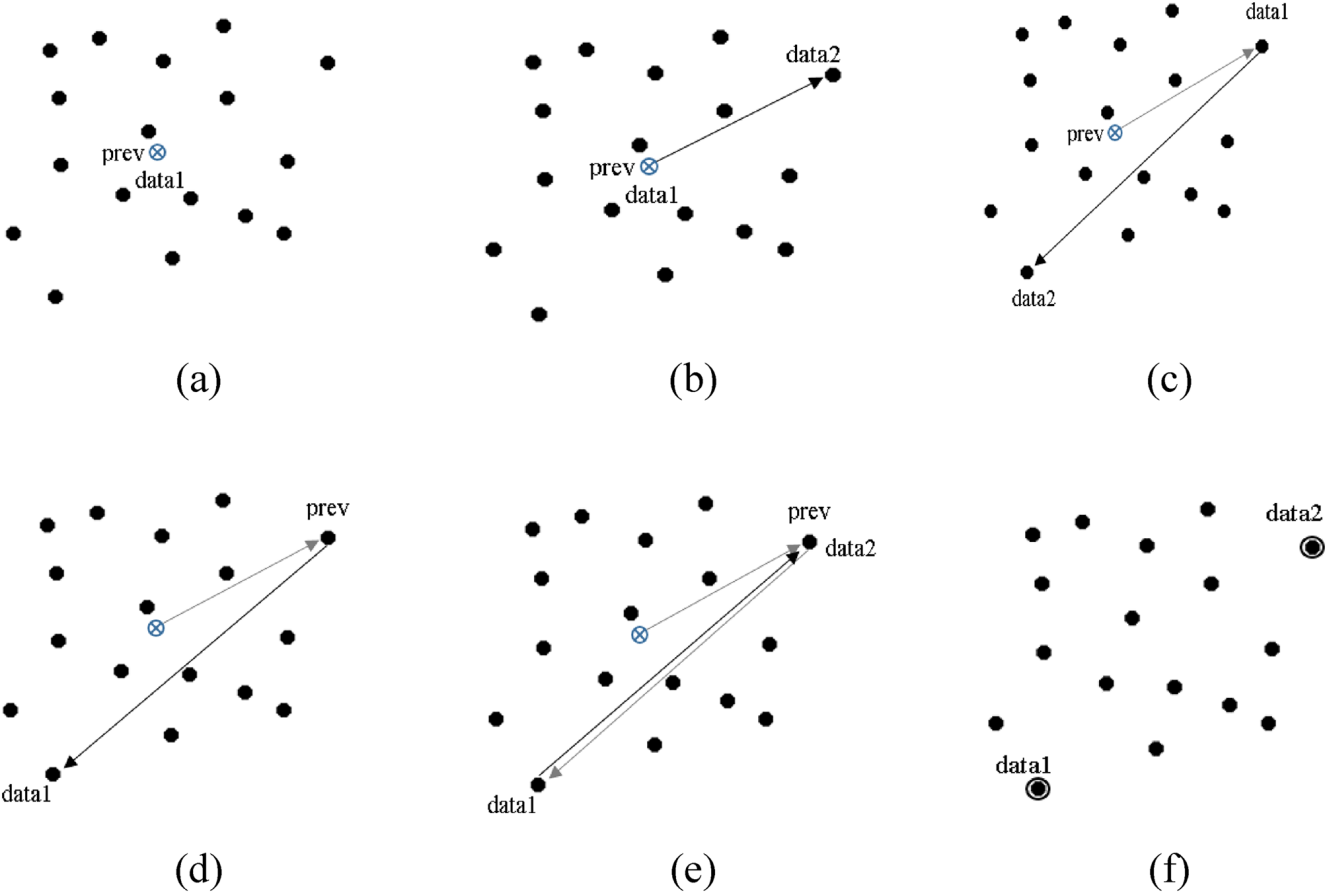
Selection of first two initial centers by discovering the farthest pair (**a**) Mean of data set is assigned as data_1_ and prev (**b**) Farthest point from data_1_ is data_2_ (**c**) Farthest point from data_2_ found and new farthest point is labeled as data_2_ and original data_2_ is labeled as data_1_ (**d**) data_1_ is labeled as prev(e) Step (c) and (d) are repeated until prev and data_2_ refers to the same data point (**f**) data_1_ and data_2_ are the farthest pair and two initial centers.

Due to the limitations of Graham Scan and Quick hull mentioned above, we came up with a new approach called FLCS, to solve the farthest pair problem. FLCS takes a greedy approach to solve this problem. Instead of traversing every data point, it only leaps to the next point which is at a maximum distance from the current point. The method stops leaping when the next farthest point is the same previous point. The pseudo code of algorithm is as follows:
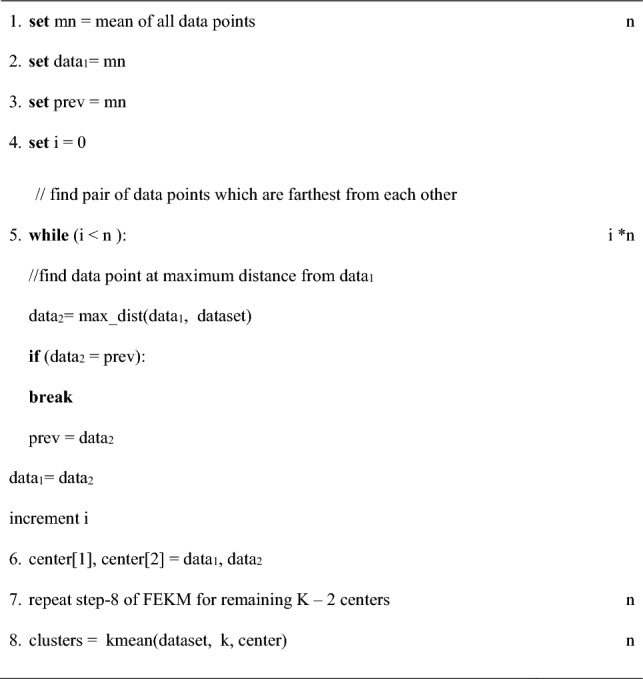


Initially, the mean of all data points is calculated and is assigned as data_1_ and prev. The farthest point from data_1_ is computed by comparing it with other data points and assigned to data_2_. Then, data_2_ is assigned to data_1_ and data_1_ is assigned to prev. Again the farthest point from data_1_ is determined and assigned to data_2_. These steps are repeated until data_2_and prev refers to the same data point. These steps are illustrated in Fig. 3. The farthest pairs data_1_ and data_2_ obtained are assigned as first two initial centers. To obtain remaining K−2 centers step-8 of FEKM is repeated.

Step 1 of the algorithm traverses all data points once to find their mean making its complexity Ɵ (n). Step 5 is used to leap from the current data point to the farthest point in order to skip redundant comparisons needed to compute the farthest pair. If we assumes number of leaps is i, then step 5 iterates i * n times. However, i is much smaller than n. So, the complexity of step5 is O(n).Then step 8 of the FEKM is used to compute remaining K−2 centers making complexity of step7 of FLCS O (n). Therefore, overall complexity of FLCS is O (n).

#### Lemma:

The number of leaps in FLCS will always be less than the total number of data points.

Given: A set of data points S, and number of leaps I.

To prove: I <|S|

#### Proof:

Let us construct a convex hull on data set S with a set of vertices V. So, there exist no data points which will be present outside the hull (Cormen^25^).

1 $${\text{and,}}|{\text{V}}| \le |{\text{S}}|^{(2/3)} \,{\text{as}}\,{\text{suggested}}\,{\text{by}}\,{\text{V}}.{\text{Jarnik}}^{28};$$

Now, there are exactly two spaces from which the method can leap.

Case I: Leaping from within the convex hull:

The farthest point from any point inside the convex hull is a vertex of the hull, as there exist no other points beyond the convex hull.

Case II: Leaping from any vertex of the convex hull:

The farthest point from a vertex of the hull will be another vertex.

By considering the above two cases it can be concluded that, once the procedure starts leaping from the first point present inside the convex hull (the first point is obtained by taking the mean of all data points), then it is not possible to leap to any other points present inside the hull. The only possibility is a leaping between the points which belongs to V.

Therefore, number of leaps, I ≤|V|≤ <${|\mathrm{S}|}^\frac{2}{3}$|S| (Hence, proved).

## Parameters for evaluation

The quality of clusters formed after the clustering process is evaluated by using few validity indices such as DI, DBI and SC. These indices measure the goodness of clusters on basis of their inter-cluster, intra-cluster distance and similarity of instances present in their created clusters. The data sets contain the class label which is used as ground truth label to compute the clustering accuracy through Rand Index. The clustering outcomes are assessed to verify if they generate perfectly homogenous and complete subgroups. For this V measure was chosen as a parameter for evaluation. The execution time of all considered methods is recorded for each of the input data sets to practically verify their efficiency.

### Validity indices

Cluster validation techniques are used to measure the quality of a cluster appropriately. Few validity indices used for this purpose are given as follows:

#### Dunn’s index (DI)

DI measure (Dunn^29^) is used to minimize the intra-cluster and maximize the inter-cluster distances. It can be defined as follows-

2 $${\rm {DI}}\left({\rm {c}} \right) { = }\mathop { {\rm {min}}}\limits_{{{\rm {i}} \in {\rm {c}}}} \left\{ {\mathop {{\rm {min}}}\limits_{{{\rm {j}} \in {\rm {c,j}} \ne {\rm {i}}}} \left\{ {\frac{{\updelta \left( {{\rm {A}}_{ {\rm {i}}}, {{\rm A}}_{ {\rm {j}}}} \right)}}{{\mathop { {\rm {max}}\left\{ {\Delta \left( { {\rm {A}}_{{\rm {k}}} } \right)} \right\}}\limits_{{{\rm {k}} \in {\rm {c}}}} }}} \right\}} \right\}$$

3 $${\text{where}},\,\delta \left( { {\text{A}}_{\rm i} , {\text{A}}_{\rm j} } \right) = \min \left\{ { {\text{d}}\left( {\underset{\raise0.3em\hbox{$\smash{\scriptscriptstyle-}$}}{ {\text{x}}} _{\rm i} ,\underset{\raise0.3em\hbox{$\smash{\scriptscriptstyle-}$}}{ {\text{x}}} _{\rm j} } \right)\left| {\underset{\raise0.3em\hbox{$\smash{\scriptscriptstyle-}$}}{ {\text{x}}} _{\rm i} \in {\text{A}}_{\rm i} ,\underset{\raise0.3em\hbox{$\smash{\scriptscriptstyle-}$}}{ {\text{x}}} _{\rm j} \in {\text{A}}_{\rm j} } \right.} \right\}$$

4 $$\Delta \left( {{\text{A}}_{\rm k} } \right) = \max \left\{ {{\text{d}}\left( {\underset{\raise0.3em\hbox{$\smash{\scriptscriptstyle-}$}}{{\text{x}}} _{\rm i} ,\underset{\raise0.3em\hbox{$\smash{\scriptscriptstyle-}$}}{{\text{x}}} _{\rm j} } \right)\left| {\underset{\raise0.3em\hbox{$\smash{\scriptscriptstyle-}$}}{{\text{x}}} _{\rm i} ,\underset{\raise0.3em\hbox{$\smash{\scriptscriptstyle-}$}}{{\text{x}}} _{\rm j} \in {\text{A}}_{\rm i} } \right.} \right\}$$

d is any given distance function used for this purpose, and A_j_ is a set consisting of the data points which are assigned to the ith cluster. Usually, any method which produces a larger value of DI determines that the cluster formed is compact and well-separated from other clusters.

#### Davies–Bouldin’s index (DBI)

DBI (Davies et al.^30^) is the ratio of the sum of data currently present within a cluster to those data remaining outside it. The data present within ith cluster distribution is given by:

5 $${\text{S}}_{{\rm i,q}} = \left( {\frac{1}{{\left| {{\text{A}}_{\rm i} } \right|}}\sum\limits_{{\underset{\raise0.3em\hbox{$\smash{\scriptscriptstyle-}$}}{{\text{x}}} \in {\text{A}}_{\rm i} }} {\left\| {\underset{\raise0.3em\hbox{$\smash{\scriptscriptstyle-}$}}{{\text{x}}} - \underset{\raise0.3em\hbox{$\smash{\scriptscriptstyle-}$}}{{\text{v}}} _{i} } \right\|_{2}^{{\text{q}}} } } \right)^{{1/{\text{q}}}}$$

The data which are between ith and jth partition is given by:

6 $${\text{d}}_{{\rm ij,t}} = \left\{ {\sum\limits_{{{\text{s}} = 1}}^{{\text{p}}} {\left| {{\text{v}}_{{\rm si}} - {\text{v}}_{{\rm sj}} } \right|^{{\text{t}}} } } \right\}^{{1/{\text{t}}}} = \left\| {\underset{\raise0.3em\hbox{$\smash{\scriptscriptstyle-}$}}{{\text{v}}} _{\rm i} - \underset{\raise0.3em\hbox{$\smash{\scriptscriptstyle-}$}}{{\text{v}}} _{\rm j} } \right\|_{\rm t}$$

where $\underline {v}_{i}$ is denoted as the ith cluster center, and both q & t are numerical values that can be chosen independently of each other and (q, t) ≥ 1. $\left| {A_{i} } \right|$ is the number of elements that are present in A_i._

Subsequently, ${\text{R}}_{{{\rm i,qt}}}$ is determined which is given by the equation:

7 $${\text{R}}_{{\rm i,qt}} = \mathop {\max }\limits_{{{\rm j} \in {\rm c,j} \ne {\rm i}}} \left\{ {\frac{{{\text{S}}_{{\rm i,q}} + {\text{S}}_{{\rm j,q}} }}{{{\text{d}}_{{\rm ij,t}} }}} \right\}$$

Finally, Davies–Bouldin’s index is obtained which is specified as follows:

8 $${\text {DB}}\left({\text {c}} \right) = \frac{1}{{\text {c}}}\sum\limits_{{{\rm i} = 1}}^{{\text {c}}} {{\text {R}}_{{\rm i,qt}} }$$

The objective of the clustering methods should focus on obtaining a minimum value of DBI for achieving proper clustering.

#### Silhouette coefficient (SC)

In SC (Rousseeuw and Silhouettes^31^), for any data point d_i_ , initially the average distance from d_i_ to all other data points belonging to its own cluster is determined, which is denoted as a. Then, the minimum average distance from d_i_ to all other data points present in other clusters are determined, which is b. Then the silhouette coefficient is calculated as follows:

9 $$\mathrm{s}=\left\{\begin{array}{l}1-\mathrm{a}/\mathrm{b },\mathrm{if\, a}<\mathrm{b}\\ 0 ,\mathrm{ if \, a}=\mathrm{b}\\ \mathrm{b}/\mathrm{a}-1 ,\mathrm{if a}>\mathrm{b}\end{array}\right.$$

The silhouette value s ranges between 0 and 1. If s is close to 1, it indicates that the sample is well-clustered, if silhouette value is almost equal to zero, it indicates that the sample lies equally far away from both the clusters, and if silhouette value is equal to -1, it indicates that the sample is somewhere in between the clusters. The number of cluster with maximum average silhouette width is taken as the optimal number of the clusters.

### Clustering accuracy

The accuracy of a cluster can be measured by using Rand Index (Rand^32^). Given N data points in a set D = {D_1_, D_2_, …, D_N_} and two clustering of them to compare C = {C_1_, C_2_, …, C_K1_} and C′ = {C′_1_, C′_2_, …, C′_K1_}, we define Rand index as:

10 $$\mathrm{R }= \frac{\mathrm{a }+\mathrm{ b}}{\mathrm{a }+\mathrm{ b }+\mathrm{ c }+\mathrm{ d}}$$

where a is number of data pairs in D that are in the same subsets of both C and C′, b is number of data pairs in D that are in different subsets of both C and C′, c is number of data pairs in D that are in same subset of C and different subsets in C′, d is number of data pairs in D that are in different subset of C and same subsets in C′. The value for R is between 0 and 1, with 0 representing two clustering do not agree on any pair of data points and 1 representing the clustering are precisely identical. Liu^33^, Guo et al.^34^, and Zou et al.^35^ investigated data collection in wireless powered underground sensor networks assisted by machine intelligence, matrix algebra in directed networks, and limited sensing and deep data mining, respectively. Shen et al.^36^, Cao et al.^37^, and Sheng et al.^38^ respectively examined the modeling of relation paths for knowledge graph completion, optimization based on mobile data, and dataset for semantic segmentation of urban scenes. Lu et al.^39^, Li et al.^40^, and Xie et al.^41^ considered multiscale feature extraction and fusion of images, patterns across mobile app usage, and a simple Monte Carlo method for estimating the chance of a cyclone impact. Recently, Liu et al.^42^, Li et al.^43^, and Fan et al.^44^ developed a multi-labeled corpus of Twitter short texts, studied the long-term evolution of mobile app usage, and proposed axial data modeling via hierarchical Bayesian nonparametric models.

### Homogeneity, completeness and V-measure

A cluster is said to be perfectly homogenous when it contains data points belonging to a single class label. Homogeneity reduces as data points belonging to different class labels are present in the same cluster. Similarly, a cluster is said to be perfectly complete if all the data points belonging to a class label are present in the same cluster. When the number of data points of a particular class label are distributed in different clusters, the completeness of the cluster decreases.

If a data set contains N number of data, cl different class labels, separated into K clusters and d data points belonging class c and cluster i then,

11 $${\text{Homogeneity}}\,{\text{is}}\,{\text{given}}\,{\text{by}}\,{\text{h}}~ = ~1{-}\frac{{{\text{F}}~\left( {{\text{cl}},{\text{K}}} \right)}}{{{\text{F}}\left( {{\text{cl}}} \right)}}$$

where F(cl, K)=$-\sum_{\mathrm{i}=1}^{\mathrm{K}}\sum_{\mathrm{c}=1}^{\mathrm{cl}}\frac{\mathrm{d}}{\mathrm{N}}\mathrm{log}\left(\frac{\mathrm{d}}{\sum_{\mathrm{c}=1}^{\mathrm{cl}}\mathrm{d}}\right)$ and F(cl) = $-\sum_{\mathrm{c}=1}^{\mathrm{cl}}\frac{\sum_{\mathrm{i}=1}^{\mathrm{K}}\mathrm{d}}{\mathrm{cl}}\mathrm{log}(\frac{\sum_{\mathrm{i}=1}^{\mathrm{K}}\mathrm{d}}{\mathrm{cl}} )$

12 $${\text{Completeness}}\,{\text{is}}\,{\text{given}}\,{\text{by}}\,{\text{c}}~ = ~1{-}\frac{{{\text{F}}~\left( {{\text{K}},{\text{cl}}} \right)}}{{{\text{F}}\left( {\text{K}} \right)}}$$

where F(K, cl) =$-\sum_{\mathrm{c}=1}^{\mathrm{cl}}\sum_{\mathrm{i}=1}^{\mathrm{K}}\frac{\mathrm{d}}{\mathrm{N}}\mathrm{log}\left(\frac{\mathrm{d}}{\sum_{\mathrm{i}=1}^{\mathrm{K}}\mathrm{d}}\right)$ and F(K) = $-\sum_{\mathrm{i}=1}^{\mathrm{K}}\frac{\sum_{\mathrm{c}=1}^{\mathrm{cl}}\mathrm{d}}{\mathrm{cl}}\mathrm{log}(\frac{\sum_{\mathrm{c}=1}^{\mathrm{cl}}\mathrm{d}}{\mathrm{cl}} )$

13 $${\text{Thus}},{\rm V} - {\text{measure}}\left( {{\text{A}}.\,{\text{Rosenberg}}\,{\text{and}}\,{\text{J}}.\,{\text{Hirschberg}},2007} \right)\,{\text{is}}\,{\text{given}}\,{\text{by}},\,{\rm V} = \frac{{\left( {1 + {\upbeta }} \right){\text{hc}}}}{{{\upbeta }{\text{h}} + {\text{c}}}}$$

where β can be set to favour homogeneity or completeness. If β is 1, then it favours both homogeneity and completeness equally. If β is greater than 1 then completeness is favoured and β less than 1 favours homogeneity.

## Results and discussion

In order to analyze the practical performance of K-Means, FEKM, MCKM, MFQ and FLCS methods, we evaluated their results on various real world data sets [UCI Repository]. The characteristics of the data set are given as follows:

A diverse range of datasets are considered to evaluate the performance of the discussed methods. These data sets in Table 1 vary in dimensions, properties and classifications. Iris data set consists of three flower classes–setosa, virginica and versicolor. It has fifty instances in each class and a total of a hundred fifty instances. Each instance has four attributes- petal length & width and sepal length & width. Similarly, seed data set consists of two hundred ten instances, seven attributes and it contains wheat kernel of three classes- Kama, Rosa and Canadian. Adult data set consists of fourteen attributes- age, work class, final weight, education, marital-status, occupation, relationship, race, sex, capital gain, capital loss, hours per week and native-country. The data set is classified into two classes- person earning more than 50 k and those with less than or equal to 50 k per year. Balance data set contains six hundred twenty-five instances of a psychological experiment. Each instance has four features which include left weight, left distance, right weight and right distance. All instances divided into three classes- scale tip to right, tip to left and balanced. Haberman is the record from study of patients who survived from surgery of breast cancer which contains a record of three hundred six patients. Patients are classified into two categories- who died within five years and who survived five years or longer. It has three attributes- age of the patient at time of operation, year of operation and number of positive auxiliary nodes detected. TAE contains teaching performance of one hundred fifty-one teaching assistances categorized into- low, medium and high as per their performances. Wine data set is the result of chemical analysis of wine growth in Italy from three different cultivators with one hundred and seventy eight samples. Each instance has thirteen features- alcohol, malic acid, ash, alkalinity of ash, magnesium, total phenols, flavonoids, no flavonoid phenols, proanthocyanins color intensity, hue, OD315 of dilute wines and proline. Mushroom data set is record of eight thousand one hundred and twenty four mushroom samples with their twenty two characteristics. The data set is classified into two categories of mushroom- edible and poisonous.

**Table 1:** Characteristics of data sets.

Datasets	No. of attributes	No. of classes	Instances present
Iris	4	3	150
Wine	13	3	178
Seed	7	3	210
Balance	4	3	625
Mushroom	22	2	8124
Abalone	8	3	4177
Glass	11	6	214
TAE	5	3	151
Adult	14	2	48,842
Haberman	3	2	306

It was discussed earlier in Section “Parameters for evaluation”(A) that, a greater value of DI or SC and a smaller value of DBI suggests better quality of cluster formation. Tables 2, 3 and 4 contains the DI, DBI and SC scores respectively where clustering loop is restricted to twenty iterations and K = 3. From these tables it can be observed that methods FEKM, MCKM, MCQ and FLCS perform better and form quality clusters than K-Means. MCQ and FLCS shows promising performance, which can be seen from the tables. In each table the best performing method is highlighted. Overall, it can be observed that for majority of the data sets for MCQ and FLCS performed better than other methods.

**Table 2:** DI score of K-means, FEKM, MCKM, MFQ and FLCS where number of iterations is 20 and K = 3.

Data sets	K-means	FEKM	MCKM	MFQ	FLCS
Iris	0.014	0.081	0.062	0.074	0.074
Balance	0.062	0.078	0.067	0.084	0.084
Abalone	0.007	0.015	0.012	0.023	0.023
Seed	0.058	0.062	0.067	0.064	0.064
TAE	0.075	0.082	0.072	0.076	0.076
Wine	0.025	0.045	0.039	0.052	0.052
Glass	0.038	0.071	0.064	0.078	0.078
Mushroom	0.061	0.075	0.069	0.079	0.079
Adult	0.066	0.073	0.079	0.072	0.072
Haberman	0.052	0.065	0.068	0.073	0.073

**Table 3:** DBI score of K-Means, FEKM, MCKM, MFQ and FLCS where number of iterations is 20 and K = 3.

Data sets	K-means	FEKM	MCKM	MFQ	FLCS
Iris	0.647	0.126	0.208	0.143	0.143
Balance	0.161	0.134	0.153	0.112	0.112
Abalone	0.796	0.578	0.624	0.499	0.499
Seed	0.348	0.205	0.167	0.191	0.191
TAE	0.175	0.118	0.183	0.132	0.132
Wine	0.52	0.439	0.501	0.346	0.346
Glass	0.812	0.147	0.204	0.134	0.134
Mushroom	0.142	0.138	0.164	0.129	0.129
Adult	0.487	0.142	0.128	0.145	0.145
Haberman	0.546	0.196	0.157	0.143	0.143

**Table 4:** SC score of K-Means, FEKM, MCKM, MFQ and FLCS where number of iterations is 20 and K = 3.

Data sets	K-Means	FEKM	MCKM	MFQ	FLCS
Iris	0.487	0.512	0.498	0.553	0.553
Balance	0.154	0.171	0.167	0.162	0.162
Abalone	0.483	0.491	0.494	0.515	0.515
Seed	0.452	0.468	0.473	0.461	0.461
TAE	0.321	0.337	0.341	0.334	0.334
Wine	0.513	0.571	0.554	0.559	0.559
Glass	0.372	0.546	0.389	0.537	0.537
Mushroom	0.234	0.259	0.248	0.281	0.281
Adult	0.552	0.542	0.546	0.559	0.559
Haberman	0.323	0.425	0.418	0.432	0.432

**Figure 4:**
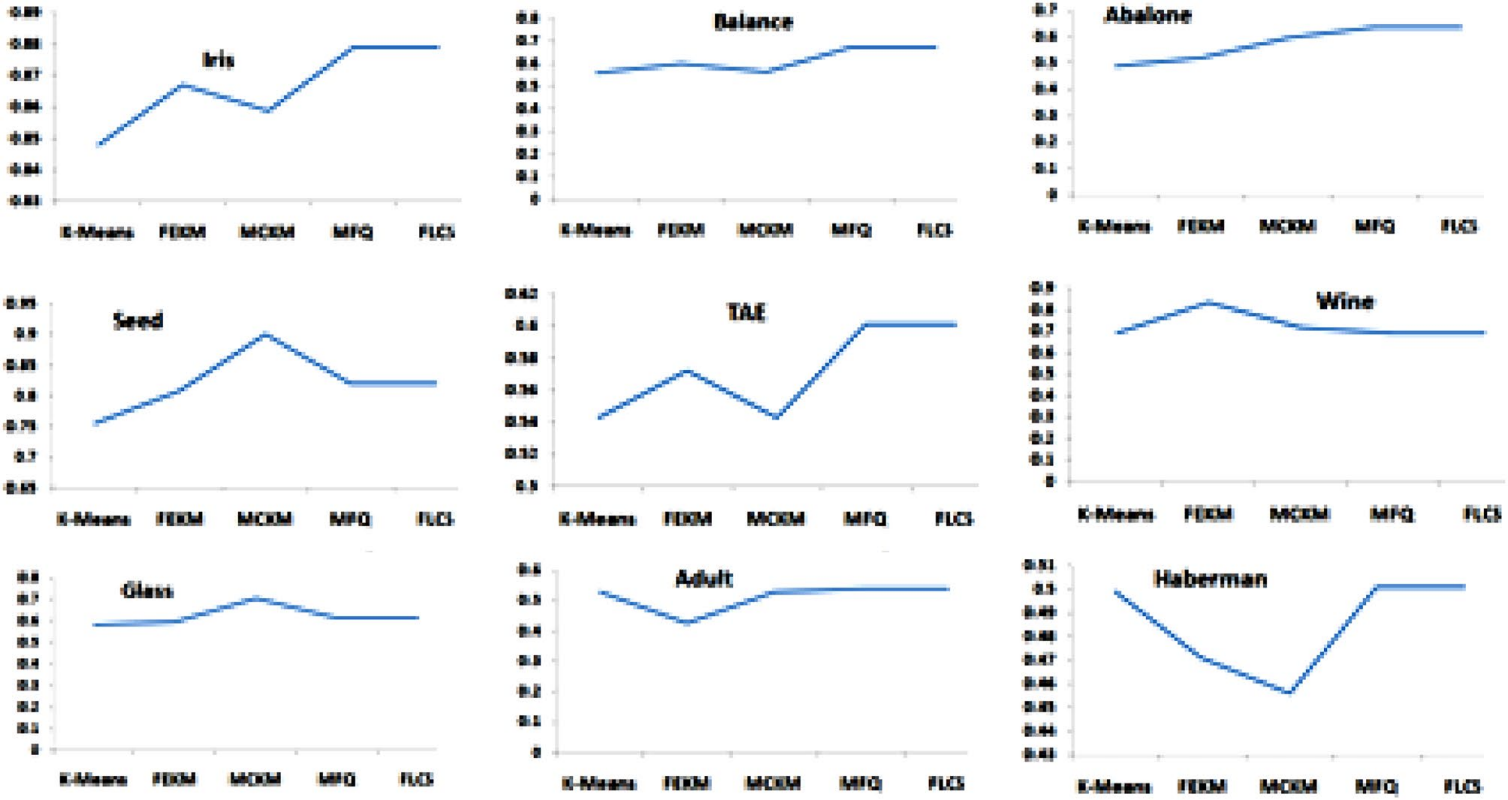
Clustering Accuracy performed on different Datasets.

The accuracy of cluster formed is the next parameter for experimental evaluation. Rand index score which was discussed in Section “Parameters for evaluation”(b), was considered for this aspect. Normally, the value of Rand Index lies between 0 and 1 and value nearer to 1 signifies better accuracy. From Table Table 6 Homogeneity score of K-Means, FEKM, MCKM, MFQ and FLCS.Data setsK-meansFEKMMCKMMFQFLCSIris0.73640.75020.73640.75140.7514Balance0.08450.15890.07770.34510.3451Abalone0.06750.05880.12090.09160.0916Seed0.66450.70750.69340.70750.7075TAE0.01950.02650.02520.01860.0186Wine0.35610.37840.42880.39870.3987Glass0.11560.12460.12130.11760.1176Mushroom0.14550.17590.16540.19350.1935Adult0.00020.000310.000280.000310.00031Haberman0.00060.00080.00080.00120.00125, the accuracy of clustering of each method can be observed on the referred data sets. MFQ and FLCS have scored better than other methods in majority of the data sets as their Rand index values are closer to 1. Only in few cases like seed, wine, glass and mushroom, FEKM and MCKM have a better Rand index value than FLCS and MCQ. Figure 4 shows the accuracy of different clustering methods performed on different datasets.

**Table 5:** Clustering accuracy score of K-means, FEKM, MCKM, MCQ and FLCS using Rand Index.

Data sets	K-means	FEKM	MCKM	MFQ	FLCS
Iris	0.848	0.867	0.859	0.879	0.879
Balance	0.561	0.602	0.566	0.674	0.674
Abalone	0.491	0.523	0.596	0.637	0.637
Seed	0.756	0.809	0.901	0.821	0.821
TAE	0.543	0.572	0.543	0.601	0.601
Wine	0.691	0.834	0.718	0.691	0.691
Glass	0.581	0.593	0.704	0.611	0.611
Mushroom	0.591	0.641	0.572	0.587	0.587
Adult	0.528	0.427	0.528	0.539	0.539
Haberman	0.499	0.471	0.456	0.501	0.501

**Table 6:** Homogeneity score of K-Means, FEKM, MCKM, MFQ and FLCS.

Data sets	K-means	FEKM	MCKM	MFQ	FLCS
Iris	0.7364	0.7502	0.7364	0.7514	0.7514
Balance	0.0845	0.1589	0.0777	0.3451	0.3451
Abalone	0.0675	0.0588	0.1209	0.0916	0.0916
Seed	0.6645	0.7075	0.6934	0.7075	0.7075
TAE	0.0195	0.0265	0.0252	0.0186	0.0186
Wine	0.3561	0.3784	0.4288	0.3987	0.3987
Glass	0.1156	0.1246	0.1213	0.1176	0.1176
Mushroom	0.1455	0.1759	0.1654	0.1935	0.1935
Adult	0.0002	0.00031	0.00028	0.00031	0.00031
Haberman	0.0006	0.0008	0.0008	0.0012	0.0012

**Table 7:** Completeness score of K-Means, FEKM, MCKM, MFQ and FLCS.

Data sets	K-means	FEKM	MCKM	MFQ	FLCS
Iris	0.7474	0.7619	0.7474	0.7649	0.7649
Balance	0.0564	0.1324	0.0648	0.2882	0.2882
Abalone	0.0721	0.0796	0.131	0.1086	0.1086
Seed	0.6681	0.7126	0.6963	0.7126	0.7126
TAE	0.0171	0.029	0.0275	0.0201	0.0201
Wine	0.3984	0.4345	0.4287	0.451	0.451
Glass	0.2234	0.2158	0.2134	0.3893	0.3893
Mushroom	0.2013	0.2218	0.2013	0.1969	0.1969
Adult	0.0002	0.000314	0.000301	0.000311	0.000311
Haberman	0.0008	0.001	0.0009	0.001	0.001

**Table 8:** V-measure score of K-means, FEKM, MCKM, MFQ and FLCS.

Data sets	K-means	FEKM	MCKM	MFQ	FLCS
Iris	0.7419	0.756	0.7419	0.7581	0.7581
Balance	0.0676	0.1445	0.0707	0.3141	0.3141
Abalone	0.0697	0.0676	0.1258	0.0994	0.0994
Seed	0.6662	0.7101	0.6949	0.7101	0.7101
TAE	0.0182	0.0277	0.0263	0.0193	0.0193
Wine	0.3760	0.4045	0.4287	0.4233	0.4233
Glass	0.1523	0.1579	0.1546	0.1807	0.1807
Mushroom	0.1689	0.1962	0.1816	0.1952	0.1952
Adult	0.0002	0.000312	0.000299	0.000317	0.000317
Haberman	0.0007	0.0009	0.0008	0.0011	0.0011

**Figure 5:**
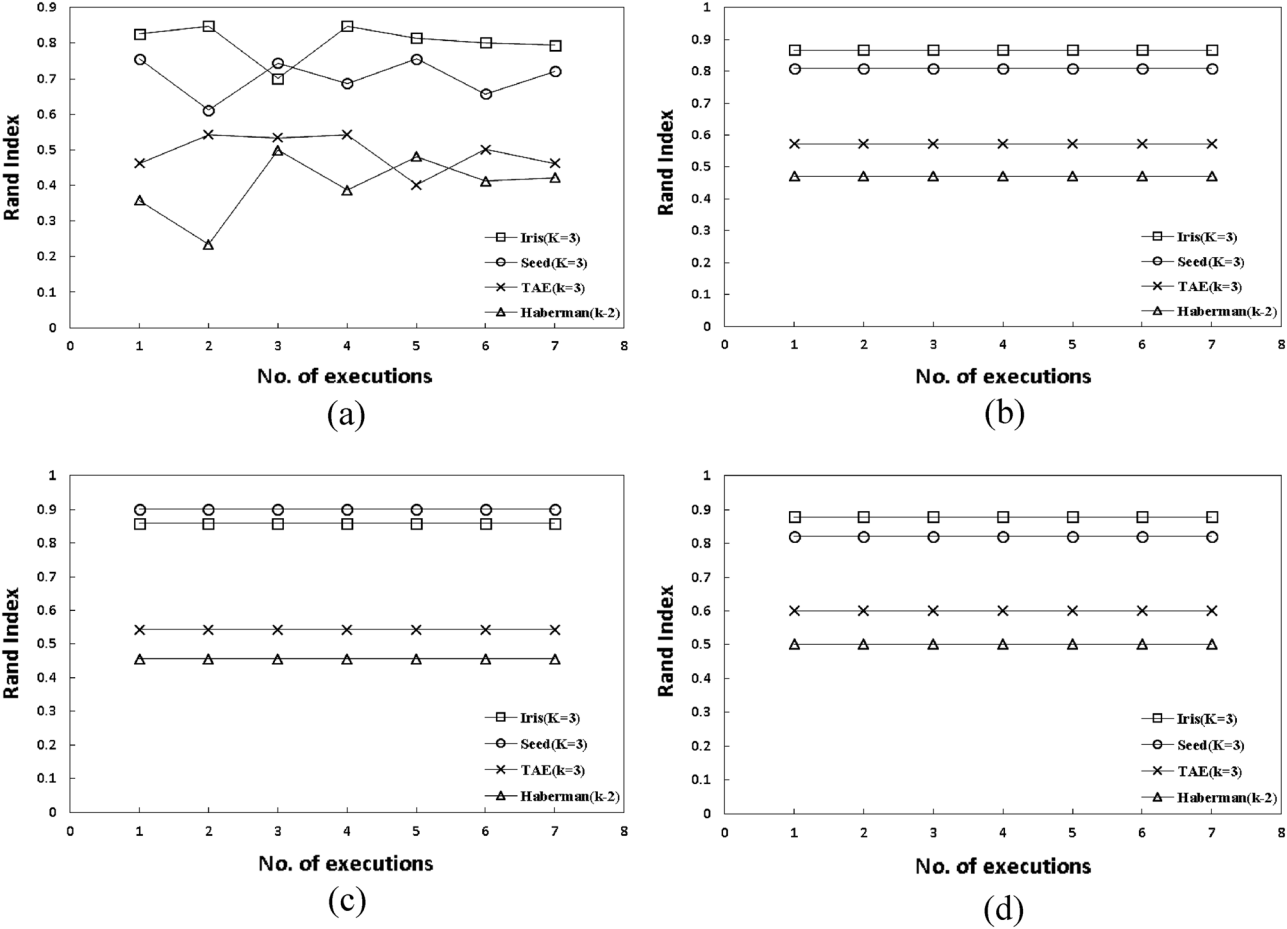
Rand index analysis for stability check performed on iris, seed, TAE and Haberman datasets (**a**) K-means, (**b**) FEKM, (**c**) MCKM, (**d**) FLCS, each executed seven times.

Next, an analysis was made to verify whether the random selection of centers as performed by K-Means will generate stable and accurate clusters or those using an approach where the centers are initially chosen by any innovative way. For better visualization, four datasets were preferred for this purpose viz, iris, seed, TAE and Haberman whose accuracy scores varies significantly. Each method including K-Means, FEKM, MCKM, MFQ and FLCS were executed seven times taking these four datasets as inputs. From Fig. 5a the unpredictability of K-Means can be clearly observed. For every execution there is a variable accuracy score Fig. 5b. Due to random initial centers, there is unpredictability in the formation of clusters due to which varied accuracy scores are generated Fig. 5c, d. This confirms that, in some cases when the random centers are chosen accurately, they produce well-organized clusters whereas in other cases, when the random centers are not precise they create malicious subgroups. Conversely, methods like FEKM, MCKM, MFQ and FLCS gives a stable and predictable output on each execution.

**Figure 6:**
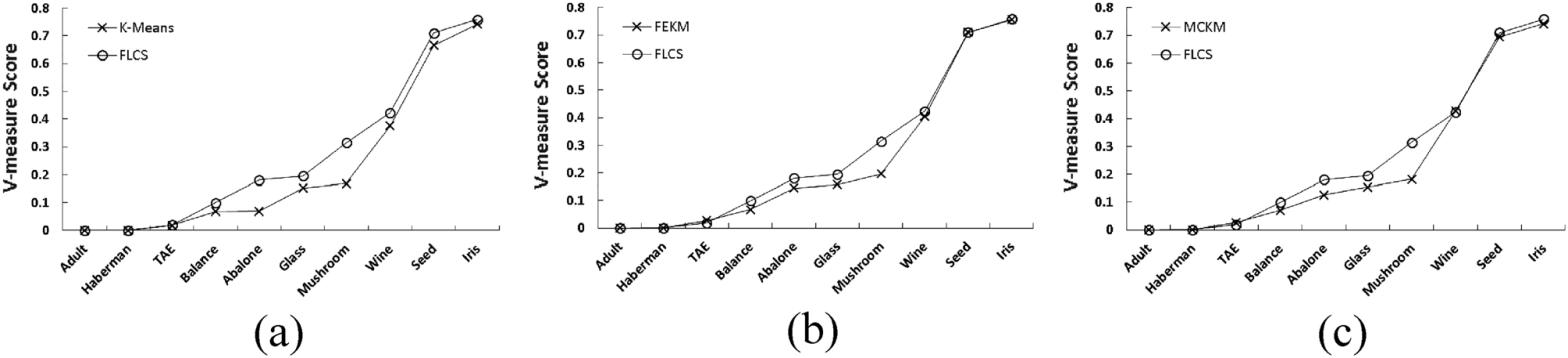
V-measure score analysis for different data sets (**a**) FLCS vs. K-means (**b**) FLCS vs. FEKM (**c**) FLCS vs. MCKM.

The following parameter for evaluation was based on determining the homogeneity and completeness of clusters using V-measure. Tables 6, 7 and 8 below illustrates these facts. For most of the data sets V-measure is closer to 1 or is on higher side for MFQ and FLCS. This implies that the clusters obtained for different data sets are precise. However, those using K-Means are not effective since the centers randomly chosen may not be the near optimal ones. Figure 6 presents an analysis of FLCS vs. K-Means, FEKM and MCKM respectively based on V-measure score for different datasets. The graph indicates V-measure score of FLCS is comparatively higher than K-Means, FEKM and MCKM which suggests that the clusters obtained with FLCS are homogenous and complete.

**Figure 7:**
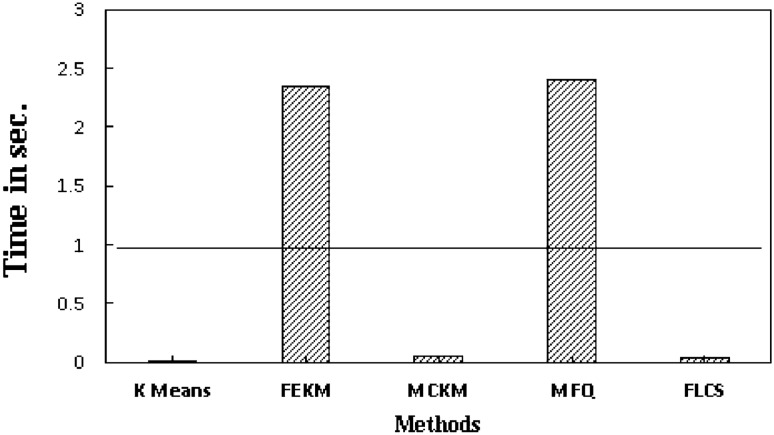
Average execution time for deciding initial centers using K-Means, FEKM, MCKM, MFQ & FLCS.

Further experiments were conducted for calculating the time taken by all the methods in determining the initial cluster centers and their convergence of clustering loop. All algorithms were executed on a machine with 5th Gen Intel^®^ i3 processor, 1.9 Ghz. clock speed and 4 GB RAM. Table 9 represents the actual execution time of all the algorithms for determining the initial centroids. From this table it can be seen that K-Means obtain the initial centers earlier than the others as this selection is done randomly. The average time taken for selection of cluster centers of each method is plotted in Fig. 7. The horizontal line in the graph represents the average time taken to select the initial centers of all the methods. From the graph it can be observed that FEKM and MFQ take slightly more time for finding the centers than the rest. MFQ by far takes the maximum out of them as its complexity increases with the increase in dimension of the data. MCKM and FLCS show similar performance, yet FLCS takes less time to compute the initial centers than MCKM due to its linear time complexity.

**Table 9:** Execution time for determining the initial centers using K-Means, FEKM, MCKM, MFQ and FLCS.

Data sets	K-means	FEKM	MCKM	MFQ	FLCS
Iris	0.000030	0.15787	0.00243	0.01866	0.00398
Balance	0.000030	3.03097	0.01759	0.01068	0.01332
Abalone	0.000039	5.01312	0.04713	5.34933	0.06515
Seed	0.000026	0.33564	0.00392	0.83359	0.00258
TAE	0.000032	0.16324	0.00243	0.02514	0.00607
Wine	0.000035	0.22144	0.00268	0.06315	0.00511
Glass	0.000028	0.35372	0.00318	1.30522	0.00266
Mushroom	0.000041	5.25642	0.07466	6.02473	0.04643
Adult	0.000052	8.23141	0.31084	10.32981	0.25333
Haberman	0.000021	0.67761	0.00275	0.01307	0.00174

**Figure 8:**
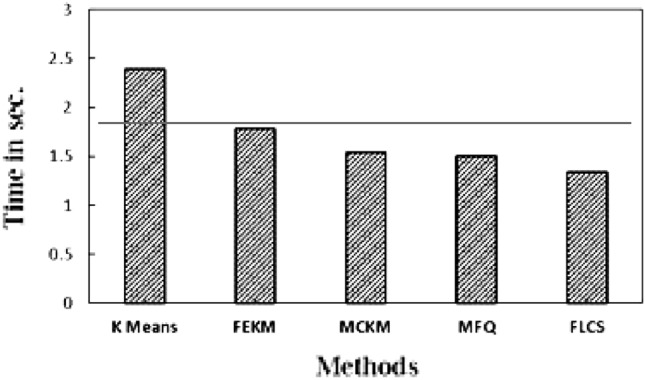
Average execution time of K-means, FEKM, MCKM, MFQ & FLCS for clustering loop convergence.

The clustering loop convergence time i.e. time taken for the cluster formation by each method on the given datasets is noted in Table 10. The best performing method on particular data set is highlighted. It can be observed from the results that, FLCS has least convergence time in five out of ten data sets. MFQ performs similarly because their initial centers are same. FEKM also have smaller convergence time in few datasets. The average clustering loop convergence time of each method is plotted in Fig. 8. The horizontal line indicates the average clustering loop convergence time of all methods. The plot shows that K-Means takes more time for formation of clusters due to bad random initial centers. K-Means is the only method above the average line. Other methods decide the subgroup formation below the average line. FLCS perform faster than other methods due to its significantly distinct initial clusters which are obtained in less time.

**Table 10:** Execution time for clustering loop convergence of K-Means, FEKM, MCKM, MFQ and FLCS.

Data sets	K-means	FEKM	MCKM	MFQ	FLCS
Iris	0.02698	0.01501	0.03621	0.01914	0.01512
Balance	0.11284	0.40028	0.11363	0.11412	0.10802
Abalone	1.43148	1.26409	1.07952	1.20457	1.21556
Seed	0.05547	0.06317	0.04802	0.02871	0.02562
TAE	0.02671	0.03224	0.09348	0.02504	0.02001
Wine	0.05622	0.02712	0.02541	0.03625	0.03849
Glass	0.16127	0.06624	0.13349	0.07648	0.07594
Mushroom	2.45328	1.94782	1.96487	2.42013	2.32307
Adult	19.48762	13.87514	11.84837	11.01519	9.51709
Haberman	0.05871	0.11392	0.03326	0.04456	0.04651

**Figure 9:**
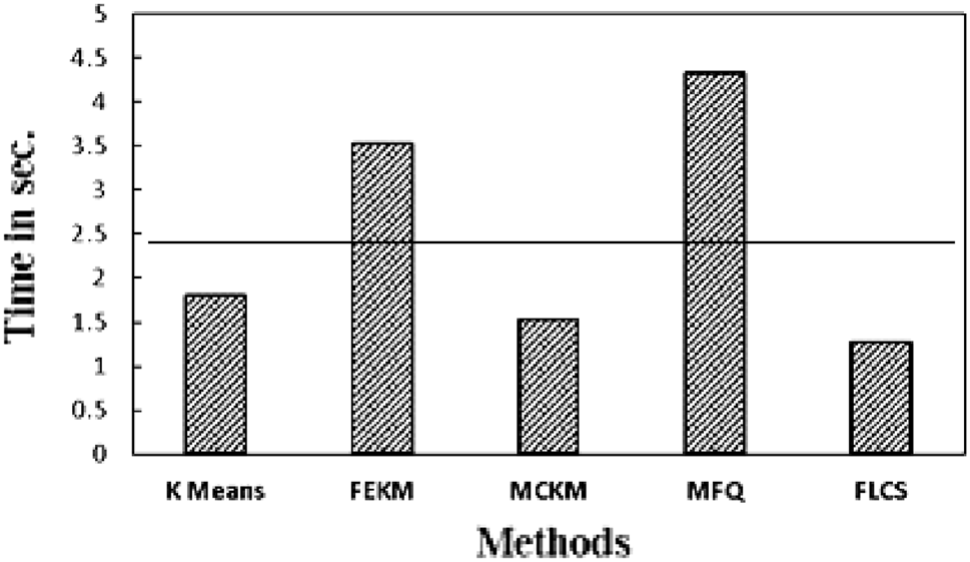
Average execution time of K-means, FEKM, MCKM, MFQ and FLCS.

Finally, overall execution time of each method employed on the given datasets is recorded. K-Means is the fastest in center selection but its random initial centers results in large convergence time. From Table 11 and Fig. 9 it can be seen that, FEKM and MFQ have overall larger execution time than others since much of their computation is spent on selecting the near-optimal centers. On the other hand, MCKM and FLCS show promising results.

**Table 11:** Actual Execution time of K-means, FEKM, MCKM, MFQ and FLCS.

Data sets	K-means	FEKM	MCKM	MFQ	FLCS
Iris	0.0271	0.0819	0.0351	0.0378	0.0205
Balance	0.3728	0.6145	0.1304	0.1248	0.1214
Abalone	1.5892	5.569	1.2447	6.554	1.4216
Seed	0.0643	0.2531	0.0559	0.8423	0.0521
TAE	0.0253	0.0964	0.0836	0.0496	0.0249
Wine	0.0521	0.0828	0.0324	0.0984	0.0459
Glass	0.1656	0.4215	0.2018	1.3924	0.1314
Mushroom	2.1354	8.0181	1.7731	8.5278	2.552
Adult	13.494	23.796	11.763	25.645	8.315
Haberman	0.0577	0.8077	0.0375	0.0576	0.0529

The performance and accuracy scores of MFQ and FLCS are more or less equal because the outcomes of the methods are always same. This is due to the fact that, both methods obtain exactly same K initial centers. However, the process of computing initial centers is totally different. From Fig. 9 difference can be clearly observed, FLCS is more efficient than MFQ in execution time.

## Conclusion

The initial centre of a cluster is a decisive factor in its final formation as erroneous centroids may results in malevolent clustering. In this research, few approaches were suggested to decide the near optimal cluster centers. FEKM was proposed with an idea to obtain well separated clusters. It was quite effective with most of the datasets, only with a complexity issue which is in the higher side. In order to get a solution to this, MCKM was suggested. It improved the computational time to some extend but still was higher than K-Means which randomly selects its centers. For these reason FCGS and MFQ were used which selects the centers present only on the convex hull thereby, trims down the chance of considering all data points to be a candidate to form the centers. However, it was found that the clustering process was effective for datasets with two or three attributes only. Due to this reason, FLCS was suggested which is simple and effective in deciding the centers with lesser complexity. All these methods were thoroughly analyzed by considering the clustering effectiveness, correctness, homogeneity, completeness, complexity and their actual execution time of convergence. For this reason performance indices like DI, DBI, and SC index were used, for correctness Rand measure was used, for homogeneity and completeness V-measure was used. All these factors considered for testing the quality of cluster formation showed excellent results for all proposed algorithms as compared to K-Means clustering. This signifies the emergence of individual groups in which data present within the group remain at a closer proximity and the separation of data of one group to that of another is very far. Zhou et al.^45^, Cheng et al.^46^ and Lu et al.^47^ investigated water depth bias correction of bathymetric LiDAR point cloud data, situation-aware IoT service coordination and IoT service coordination using the event-driven SOA paradigm respectively. Quantifiable privacy preservation for destination prediction in LBSs, spatio-temporal analysis of trajectory data and energy-efficient framework for internet of things underlaying heterogeneous small cell networks respectively examined by Refs.^48–50^. Peng et al.^51^, Bao et al.^52^, Liu et al.^53^ recently examined the community structure in evolution of opinion formation, limited real-world training data and robust online tensor completion for IoT streaming data recovery respectively. Liu et al.^54^ worked on federated neural architecture search for medical sciences. Refs.^55–57^ exhibits the recent and newly development in the IoT field like next-generation wireless data center network, fusion network for transportation detection and wireless sensor networks with energy harvesting relay.

This work guided us to quite a few exciting and innovative research opportunities which can be further explored viz, both the time complexity and computation time of the suggested methods can be further reduced, the concept of convex hull can still be properly used to obtain better cluster centers on versatile data sets.

## Data Availability

The data will be made available on a reasonable request to the corresponding author.
